# The Influence of the Coach’s Autonomy Support and Controlling Behaviours on Motivation and Sport Commitment of Youth Soccer Players

**DOI:** 10.3390/ijerph18168699

**Published:** 2021-08-17

**Authors:** Javier Sevil-Serrano, Ángel Abós, Sergio Diloy-Peña, Pedro L. Egea, Luis García-González

**Affiliations:** 1 Faculty of Education, University of Extremadura, Av. de Elvas, s/n, 06006 Badajoz, Spain; jsevils@unex.es; 2 Faculty of Social Sciences and Humanities, EFYPAF “Physical Education and Physical Activity Promotion” Research Group, University of Zaragoza, C/Atarazanas 4, 44003 Teruel, Spain; aabosc@unizar.es; 3 Faculty of Health and Sport Sciences, EFYPAF “Physical Education and Physical Activity Promotion” Research Group, University of Zaragoza, Plaza Universidad, 3, 22002 Huesca, Spain; sdiloy@unizar.es; 4 Faculty of Health and Sport Sciences, University of Zaragoza, Plaza Universidad, 3, 22002 Huesca, Spain; pedroegea87@hotmail.com

**Keywords:** adolescents, soccer, self-determination theory, coach, motivation, motivating style, controlling use of rewards, intimidation, excessive personal control, sport commitment

## Abstract

The coach is one of the most influential agents in the sport commitment of youth players. Grounded in self-determination theory (SDT), numerous studies have examined the influence of the coach’s autonomy-supportive behaviours on athletes’ motivation. However, fewer studies have examined the influence of the coach’s controlling behaviours. The aim of this cross-sectional study was to analyse the influence of young soccer players’ perception of their coach’s autonomy-supportive and controlling behaviours on the satisfaction and frustration of their basic psychological needs (BPN) and sport commitment. A total of 203 soccer players (86% boys), aged 10–19 years (*M* = 14.88; *SD* = 1.54) participated. Coach autonomy support positively predicted BPN satisfaction which, in turn, positively explained sport commitment. Coach intimidation behaviours positively predicted BPN frustration, which, in turn, negatively explained sport commitment. In cross-relationships, autonomy support negatively explained BPN frustration, while intimidation behaviours and the controlling use of rewards negatively predicted BPN satisfaction. To conclude, these results suggest that it is important for the coach not only to support autonomy, but also to avoid the use of controlling behaviours, especially intimidation and controlling use of rewards, because of their influence on the motivational processes and sport commitment of youth soccer players.

## 1. Introduction

Organised sport, such as soccer, represents an opportunity for young people to increase their physical activity (PA) levels [1]. Yet, a quarter of youth category soccer players drop out of this sport each year [2]. Consequently, promoting positive experiences in youth soccer players seems a key aspect to obtain all the benefits derived from this sport [3]. Conversely, negative experiences in this context can lead to early sport drop-out [4]. At this point, the figure of coaches may play a key role in the athlete’s learning process, and can be a positive influence on the motivation and sport commitment of young soccer players [5,6,7].

Self-determination theory (SDT) [8,9] is one of the most widely used theoretical frameworks within the sport context to explain the motivation of athletes in the sport context [10]. This theoretical framework [8] indicates that there are three basic psychological needs (BPN; i.e., autonomy, competence, and relatedness), which are essential psychological nutrients to enhance self-determined motivation and, consequently, to develop positive and more adaptive consequences in athletes [9]. In the sport context, athletes satisfy autonomy when they feel that they are the source of their own actions. Similarly, when they feel that they are effective or skilled in the proposed activities, they satisfy their competence need. Finally, athletes satisfy the relatedness need when they have satisfactory interpersonal relationships and are integrated with their teammates [9]. 

In contrast, the frustration of these BPN may lead athletes towards less self-determined motivation, triggering more negative and maladaptive consequences [11], even leading to sport drop-out. Specifically, autonomy is frustrated when players perceive pressure and alienation in the activities they perform. Competence is frustrated when athletes experience a sense of failure or incapacity to successfully complete the proposed tasks. Finally, relatedness is frustrated when players feel isolated and rejected within the team [9]. A vast body of research in the sport context [12,13,14,15,16] has shown that BPN satisfaction and frustration are two related but independent constructs. In this sense, prior evidence has revealed that both BPN satisfaction and frustration may be associated with different antecedents and outcomes [14,16,17]. In addition, two recent person-centred studies have also shown that even some athletes may experience simultaneously need satisfaction and need frustration [13,15]. To illustrate, athletes might perceive satisfaction (e.g., “I feel that I can choose between two tasks”) and frustration (e.g., “I feel that I am restricted to behave in a certain way”) of their autonomy in the same training session [15]. Consequently, it seems required that new research in the sport context jointly examines BPN satisfaction and frustration to better understand the athletes’ motivational experience.

SDT [8] understands people as active organisms that can interact with the social environment. In this sense, there are a series of agents that are indispensable both to satisfy BPN and to avoid their frustration. Thus, one of the most influential social agents in the motivational process of players is the coach [5,6,7]. In the sport context, coaches can support autonomy by using more democratic teaching styles, where players take more responsibility and makes their own decisions [18]. Diverse studies in the youth sport context have shown that young soccer players who perceive an autonomy-supportive style from their coaches satisfy their BPN to a greater extent and, in addition, have less frustration with their BPN [19,20,21].

On the other hand, coaches may also adopt a controlling style to make their athletes think and act in a prescribed manner. Previous studies [22,23] have identified that this controlling style may be composed of four conceptually distinct controlling behaviours: controlling use of rewards, negative conditional regard, intimidation, and excessive personal control. Controlling use of rewards refers to the use of tangible and verbal rewards to incentivise players’ participation and goal achievement. Negative conditional regard refers to a coach’s withdrawal of attention and affection from their players when they do not behave in the way they expect. Intimidation behaviours, such as verbal abuse, threats, or the use of physical punishment, are used in order to humiliate and belittle players. Finally, controlling behaviours with excessive personal control are those that interfere in areas of players’ private lives that are not directly associated with the sport they play [22]. Different studies in the sport context have pointed out that youth players who perceive a controlling style from their soccer coaches are more likely to experience BPN frustration [21,24]. However, there are a limited number of studies that have examined the relationship of the four controlling coach behaviours with BPN satisfaction and frustration, especially in youth soccer players. A study [25] with U-15 and U-18 soccer players indicated that all four controlling behaviours were negatively associated with relatedness and competence satisfaction, with the exception of negative conditional regard in the need for competence. However, no controlling behaviour was related to autonomy satisfaction in that study. In another study conducted with young soccer players [26], the coach’s excessive personal control negatively predicted athletes’ autonomy satisfaction and positively autonomy frustration. Furthermore, the same study [26] showed that the coach’s negative conditional regard negatively predicted athletes’ competence and relatedness satisfaction and positively predicted frustration of both needs. This limited number of studies and the discrepancies in the results found raise the need to further examine the relationship of different controlling behaviours on both BPN satisfaction and frustration in youth soccer players. Knowing which controlling behaviours are associated with more maladaptive consequences in players may be of great interest to coaches in order to avoid or reduce their use both in training and in matches.

Finally, SDT [9] indicates that, depending on the satisfaction or frustration of BPN, different affective, cognitive and behavioural consequences can be triggered. One of these possible behavioural consequences is sport commitment, defined as a psychological construct that represents the desire and decision to continue practicing sport [27]. Different research in the sport context has evidenced that soccer players who report higher levels of BPN satisfaction have higher sport commitment [28,29]. Conversely, players with higher levels of BPN frustration tend to have lower sport commitment [28,29].

### The Present Study

To the authors’ knowledge, there are numerous studies in the sport context, and specifically in the field of youth soccer, that have examined the influence of coach autonomy support on BPN satisfaction, and players’ sport commitment (i.e., the “bright side” of motivation). However, fewer studies have examined the influence of different coach’s controlling behaviours on BPN frustration (i.e., the “dark side” of motivation). Moreover, a very limited number of studies have examined the cross-relationships between autonomy support and BPN frustration, as well as between different controlling behaviours and BPN satisfaction. Therefore, further studies, jointly examining the bright and the dark sides sequence postulated by SDT, seem to be needed.

**Figure 1:**
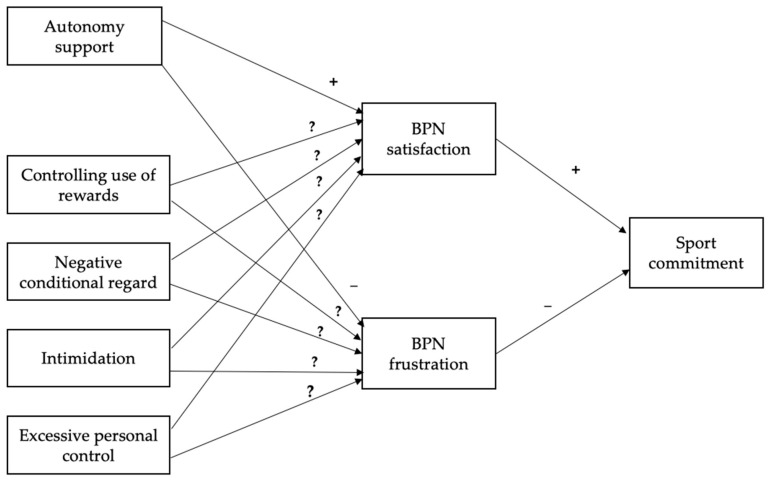
Hypothesised predictive relationships between coach motivating styles and sport commitment, through BPN satisfaction and frustration. BPN = Basic psychological needs.

To fill this research gap, the aim of the study was to analyse the influence of youth soccer players’ perception of coach autonomy support and controlling behaviours on BPN satisfaction, BPN frustration, and sport commitment. In line with previous studies [19,20,21], we hypothesised that players’ perceived autonomy support would predict both BPN frustration and satisfaction, and the latter would predict their sport commitment. Yet, given the disparity of previous results in youth soccer players [25,26], the relationship of the four controlling behaviours (i.e., controlling use of rewards, negative conditional regard, intimidation, and excessive personal control) with BPN satisfaction and frustration was not specifically hypothesised. Nevertheless, we expected that athletes’ BPN frustration would negatively predict sport commitment (Figure 1).

## 2. Materials and Methods

### 2.1. Design and Participants

A total of 203 soccer players (86% boys and 14% girls), aged between 10 and 19 years (*M* = 14.88; *SD* = 1.54), from two clubs in the region of Huesca, participated in this cross-sectional study. Of the 203 players, 84 belonged to the under-14 category (U-14), 94 belonged to the under-16 category (U-16) and, finally, 25 belonged to the under-18 category (U-18). The average experience in soccer was 7.09 years (*SD* = 2.50).

### 2.2. Measures

In the present study, the instruments stems, which had been validated in the sport context, were slightly adapted to soccer (e.g., “When I do sport…” was changed to “When I play soccer…”). The measured variables were the following:

#### 2.2.1. Perceived Coach Autonomy Support

Athletes’ perceptions of their coaches’ autonomy support were assessed using the Spanish version of the Sport climate questionnaire (S-SCQ) [30] in its reduced, 6-item version (e.g., item #3: “My coach offers me different alternatives and options”). Responses to each of the items were collected on a 7-point Likert-type scale, ranging from 1 (“strongly disagree”) to 7 (“strongly agree”). In the current study, the confirmatory factor analysis (CFA) showed a good fit to the data (χ^2^(9) = 10.266, *p* < 0.33; CFI = 0.99; TLI = 0.99; RMSEA = 0.03; RMSEA = 0.03).

#### 2.2.2. Perceived Coach Controlling Behaviours 

Athletes’ perceptions of their coaches’ controlling behaviours were measured using the Spanish version of the Controlling coach behaviours scale (CCBS) [24]. The CCBS includes 15 items that assess controlling use of rewards with four items (e.g., item #14: “The only reason my coach rewards/praises me is to make me train harder”), negative conditional regard with four items (e.g., item #8: “My coach pays me less attention if I have displeased him/her”), intimidation with four items (e.g., item #2: “My coach shouts at me in front of others to make me do certain things”), and excessive personal control with three items (e.g., item #5: “My coach tries to control what I do during my free time”). Responses were registered on a 7-point Likert scale ranging from 1 (“totally disagree”) to 7 (“totally agree”). In the current study, the CFA showed adequate fit to the data (*χ*^2^(84) = 193.059, *p* < 0.01; CFI = 0.90; TLI = 0.89; RMSEA = 0.08).

#### 2.2.3. BPN Satisfaction

Athletes’ BPN satisfaction was measured using the Spanish version of the Motivating mediators in sports scale [31]. This scale includes 23 items introduced by the stem “When I play soccer...”, which assess competence satisfaction with seven items (e.g., item #4: “I feel that I am among the most capable”), autonomy satisfaction with eight items (e.g., item #5: “I feel I can make my own decisions”), and relatedness satisfaction with eight items (e.g., item #7: “I feel good with the people I train with”). Responses were registered on a 5-point Likert scale ranging from 1 (“totally disagree”) to 5 (“totally agree”). In this study, the second order-factor CFA showed adequate fit to the data (*χ*^2^(206) = 935.778, *p* < 0.01; CFI = 0.911; TLI = 0.904; RMSEA = 0.076).

#### 2.2.4. BPN Frustration

Athletes’ BPN frustration was measured using the Spanish version in sport context of the Psychological need thwarting scale (PNTS) [32]. This scale is composed of 12 items, with 4 items per factor, which assess competence frustration (e.g., item #6: “I feel frustrated because I am not given opportunities to fulfil my potential”), autonomy frustration (e.g., item #5: “I feel obliged to follow training decisions made for me”), and relatedness frustration (e.g., item #10: “ I feel other people dislike me”). Responses were registered on a 7-point Likert scale ranging from 1 (“totally disagree”) to 7 (“totally agree”). In this study, the second-order factor CFA revealed adequate fit to the data (*χ*^2^(51) = 110.175, *p* < 0.01; CFI = 0.93; TLI = 0.91; RMSEA = 0.07).

#### 2.2.5. Sport Commitment

Athletes’ sport commitment was measured using the Spanish version of the Sport commitment questionnaire (SCQ) [33]. Of the 28 items that comprise the SCQ, in the present study, only the 6 items that assess sport commitment (e.g., item #5: “I am determined to continue playing soccer next season”) were used. Responses were registered on a 5-point Likert scale ranging from 1 (“totally disagree”) to 5 (“totally agree”). In this study, the CFA showed adequate fit to the data (*χ*^2^(5) = 21.435, *p* < 0.01; CFI = 0.91; TLI = 0.90; RMSEA = 0.08).

### 2.3. Procedure

First, soccer teams in the Huesca region that might be interested in participating in the study were contacted. With the aim of interested soccer clubs to know the purpose of the study, an information dossier was provided. With their acceptance, and once the club coordinators and coaches had been informed, parental authorisation was requested for under-age players, and the signature of the informed consent form for over-age players. The process of completing the questionnaires was carried out in a paper-and-pencil format before the training sessions, in group (i.e., at the same time with teammates), in the presence of the principal investigator, and in the absence of the coaches of the two teams, so as not to condition the answers. Data collection lasted approximately 30 minutes and took place in the changing rooms. Beforehand, the participants were informed that the data were confidential and anonymous and would only be used for research purposes. The guidelines of the Declaration of Helsinki, regarding compliance with ethical standards in research, were respected throughout the process. 

### 2.4. Data Analysis

First, a composite reliability analysis of the variables was carried out using the Omega coefficient [34]. Next, descriptive statistics—i.e., means (M) and standard deviation (SD)—and bivariate correlations between the study variables, through Pearson’s correlation coefficient, were calculated. These analyses were carried out with SPSS 23.00 software program. Finally, a factor score path analysis was used to test relationships between the study variables, following theoretical tenets of SDT. Factor score path analysis is considered an alternative for structural equation modelling (SEM), because it does not require large sample sizes in complex models, and overcomes misspecification errors with small sample sizes [35]. In a first step, factor scores were calculated by performing a factor analysis and calculating factor scores for each variable with Mplus version 8.0. (Los Angeles, CA, USA) Factor scores were estimated for the true latent variable scores. In a second step, factor scores were used for path analysis through linear regression, as if they were the true latent variable scores when using a SEM [35]. The factor score path analysis model was performed via maximum likelihood robust estimator (MLR). To evaluate the model fit, the comparative fit index (CFI), the Tucker–Lewis index (TLI), and the root mean square error of approximation (RMSEA) were selected. Higher values of 0.90 and 0.95 for CFI and TLI indicate good and excellent fit, respectively. Likewise, values of 0.08 and 0.06 or less for RMSEA indicate adequate and excellent fit, respectively [36].

## 3. Results

### 3.1. Descriptive Statistics, Reliability, and Correlations between the Study Variables

Descriptive statistics (M and SD), reliability values and bivariate correlations between the study variables are reported in Table 1. Following the sequence established by the bright side of the SDT, it was shown how perceived coach autonomy support was positively and significantly related to BPN satisfaction (*r* = 0.529), as well as negatively and significantly related to BPN frustration (*r* = −0.453). Similarly, BPN satisfaction is positively and significantly related to sport commitment (r = 0.488). In contrast, following the sequence established by the dark side of the SDT, all controlling behaviours (i.e., controlling use of rewards, negative conditional regard, intimidation, and excessive personal control) negatively and significantly correlated with BPN satisfaction (r = −0.169 to −0.306), as well as positively and significantly with BPN frustration (r = 0.412 to 0.646). BPN frustration negatively and significantly correlated with sport commitment (r = −0.350). 

### 3.2. Factor Scores Path Analysis

**Figure 2:**
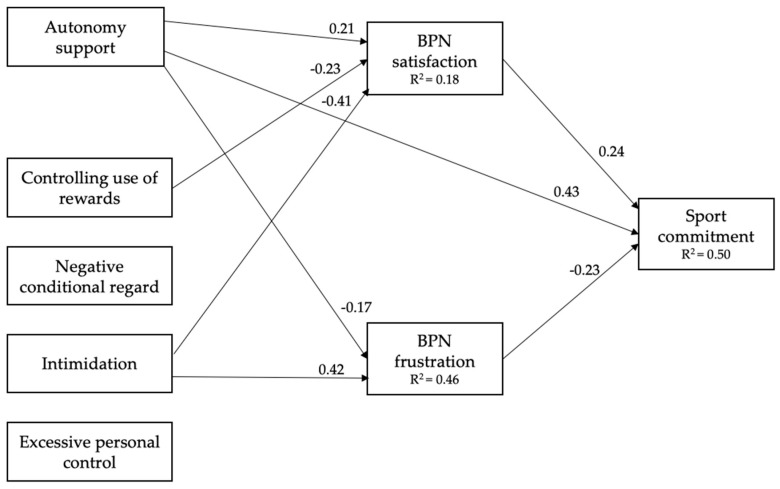
Predictive relationships between students’ perceptions of coach motivating styles, and sport commitment, through BPN satisfaction and frustration. BPN = Basic psychological needs. To gain clarity in the interpretation of the model, only significant relationships are shown.

Figure 2 presents the tested factor score path analysis, which obtained a good fit with the data: *χ*^2^ = 7.054, *p* = 0.133; *χ*^2^/df = 1.76; CFI = 0.987; TLI = 0.943; SRMR = 0.040; RMSEA = 0.061 (90% CI = 0.000–0.134). Autonomy support positively predicted BPN satisfaction and sport commitment, and negatively predicted BPN frustration. With regard to the four controlling behaviours, the controlling use of rewards negatively predicted BPN satisfaction. In addition, personal intimidation negatively predicted BPN satisfaction and positively predicted BPN frustration. Sport commitment was positively explained by BPN satisfaction and negatively by BPN frustration. The model accounted for 50% of the variance in sport commitment. 

In addition to the direct effects shown in Figure 2, Table 2 reports the indirect effects between the athletes’ perceptions of coaches’ motivating styles and sport commitment. The results showed how both need-based experiences mediate the relationship between autonomy support and sport commitment. These mediation effects of athletes’ need-based experiences were the same between coach intimidation and sport commitment. In contrast, the relationship between the controlling use of rewards and sport commitment was only mediated by BPN frustration.

## 4. Discussion

Grounded in SDT, while most studies in young players have examined the influence of coach autonomy support on athletes’ BPN satisfaction, research that has analysed the influence of coach’s controlling behaviours on athletes’ need-based experiences is scarcer. Moreover, the cross-relationships between the bright and dark side of motivation variables have been little explored. With the aim of expanding previous knowledge, the present study, in addition to examining the role of youth soccer players’ perceived autonomy support from their coaches, also examined the influence of the coaches’ controlling behaviours (i.e., controlling use of rewards, negative conditional regard, intimidation, and excessive personal control) on players’ need-based experiences and sport commitment. The evidence obtained from the present study may be useful to coaches in order to improve the motivational process and sport commitment of young soccer players, avoiding high drop-out in this sport [2].

Consistent with our hypothesis, and in line with SDT [8] and previous studies on young soccer players [21,37], the results of this study showed how coach autonomy support is a key factor in satisfying athletes’ BPN. In this sense, if, during training sessions and matches, the coach makes it easy for players to take their own decisions, this could help players to perceive their needs for autonomy, competence, and relatedness as satisfied. More particularly, for example, if players can participate in choosing strategies for the team, they will feel that the actions derive from themselves (i.e., autonomy satisfaction) [21]. Similarly, if players can choose the type of tasks, or the level of difficulty of exercises in training, they will be able to feel that they are effective and making progress (i.e., competence satisfaction) [21]. Finally, if they can also participate in choosing the groups, they will be able to feel more integrated with their peers (i.e., relatedness satisfaction) [21]. 

Furthermore, in line with SDT [8] and with previous studies on youth soccer players [19,20], the results showed a cross-relationship between the coach’s autonomy support and athletes’ BPN frustration. These results were also in line with our hypothesis. In this sense, it seems that encouraging the use of autonomy-supportive strategies by the coach, in addition to facilitating BPN satisfaction, may act as a protective factor against BPN frustration [38]. Finally, our results also showed a direct relationship between autonomy support and sport commitment, an association that had not been shown in previous studies in youth soccer players [28,39]. In this sense, this is the first study in a sport context with youth players to demonstrate how coach autonomy support can directly facilitate sport commitment. Therefore, it seems necessary for coaches to implement autonomy support strategies where players feel that they have opportunities to take their own decisions, as this may influence the motivational process, and sport commitment, through greater satisfaction and less frustration of their three BPN [20,21].

On the other hand, in line with SDT [11], the results of the correlations analysis showed positive and significant associations between the four coach control behaviours (i.e., controlling use of rewards, negative conditional regard, intimidation, and excessive personal control) and BPN frustration. However, in the factor score path analysis, only intimidation behaviour was found to positively and significantly predict BPN frustration. Regarding this part of the study’s aim, no hypotheses were formulated since previous studies did not provide clear evidence. Nevertheless, our results are in line with the study of Moreno-Luque and colleagues [25], where it was also found that intimidation by the coach frustrated the BPN of youth soccer players. In this vein, it seems that when players feel belittled or humiliated by the coach (e.g., verbal abuse or threats), they may feel their BPN frustrated. For example, if a coach uses verbal abuse to impose actions on his or her players, it may lead to pressure and alienation of the athlete (i.e., autonomy frustration) [26]. Similarly, if players receive threats for failing an action, or are punished after a failure, this could generate feelings of inferiority (i.e., competence frustration) [25]. Finally, if a coach publicly and repeatedly threatens or punishes some players and not others, this may generate comparisons between them, making some athletes feel isolated and poorly integrated (i.e., relatedness frustration) [25]. 

Yet, in the present study, the other three control behaviours (i.e., controlling use of rewards, negative conditional regard, and excessive personal control) of the coach did not significantly predict players’ BPN frustration. As suggested by previous studies in athletes [26,40,41], the presence of controlling behaviours by coaches is often commonplace, which, despite the negative consequences with which they have been associated, may become normalised by players from an early age [42]. This may cause the explanatory effects of controlling use of rewards, negative conditional regard, and excessive personal control towards BPN frustration to be diluted in the present study. Nevertheless, as mentioned above, the correlational results found in the present study, in line with previous studies [43], suggest that controlling behaviours are negative for athletes’ motivational development and sport commitment. 

On the other hand, with respect to cross-relationships, the results, in line with Cano and colleagues [44], showed that the controlling style provided by the coach, in addition to frustrating BPN, can also undermined BPN satisfaction. More specifically, the results of the model showed a negative relationship between intimidation and the controlling use of rewards with BPN satisfaction. In this sense, it seems that if a coach only manages rewards to incentivise participation and goal achievement, linked to the achievement of successful actions (i.e., controlling use of rewards), those players who do not receive them will have a lower feeling of efficacy and progress (i.e., lower competence satisfaction) [44]. Similarly, if some players perceive that they cannot participate in the choice of such rewards provided by the coach, they may have the perception that they are not the source of their actions (i.e., lower autonomy satisfaction). Finally, if in addition, some players perceive that these rewards are distributed differently among the team; it is possible that they form less satisfactory interpersonal relationships, and they even feel less integrated with their teammates (i.e., lower relatedness satisfaction) [44]. Therefore, although not all behaviours that make up the control style are perceived in the same way [23,45], the findings of this study highlight how the presence of controlling behaviours, especially intimidation and the controlling use of rewards, are not positive for the motivational development and commitment of young soccer athletes [43,46]. Therefore, coaches should avoid, or at least reduce, controlling behaviours, especially intimidation and controlling use of rewards, to ensure that athletes do not frustrate their BPN. 

In addition, in line with SDT [8] and with the hypothesis stated, the findings of the factor score path analysis showed a positive relationship between BPN satisfaction and sport commitment. The results show how satisfying these psychological mediators plays a fundamental role in obtaining high levels of sport commitment in players. These results are in line with previous studies on youth soccer players [29] which also showed that satisfying the three BPN significantly and positively predicted sport commitment. These results may be due to the fact that when players feel that they are the source of their actions, they perceive themselves as important and skilled within the team, and they feel that they form part of a group, they are more committed to the sport [47]. On the other hand, the present study, in line with previous studies [28] and with the stated hypothesis, also showed how BPN frustration negatively predicted sport commitment. In this sense, it seems that BPN frustration could trigger lower player commitment [48]. Thus, a player who perceives that his or her actions are imposed by his or her coach, who does not feel competent in training and matches, and who may not feel integrated with his or her teammates, is likely to have less desire to engage in the sport [48]. This reinforces the importance of coaches supporting autonomy, and reducing controlling behaviours with their players, in order to promote the satisfaction of these BPN, as well as to avoid their frustration.

Although the findings expand on previous evidence and underline the importance of coaches’ motivational styles, it is also important to point out the limitations and some prospects of the present study. Firstly, the sample used was purposive. Furthermore, the age difference between the participants (i.e., from 10 to 19 years old) could have caused a bias in the understanding of the items, so the results should be interpreted with caution. Future studies should use probability sampling to increase the external validity of the results. In addition, the data collected only focused on the characteristics of the young soccer players. Future research should take into account socio-demographic data of coaches (e.g., age, experience, gender, etc.) to control the results. Secondly, a cross-sectional design was used, so causal relationships between the study variables cannot be inferred. In order to obtain more rigorous scientific evidence on the consequences of the coach’s motivational style on the motivational process, longitudinal studies are needed. Moreover, quasi-experimental studies based on increasing coach autonomy support and reducing controlling behaviours would be necessary to test their effect on BPN satisfaction and frustration, as well as the sport commitment of youth soccer players. Thirdly, only sport commitment was included as a positive outcome in the present study. Future studies should evaluate other negative consequences, such as sport drop-out, to get a more complete picture of the bright and dark side of players’ motivation. Fourthly, only the players’ perception of the coach’s autonomy support and controlling behaviours was examined by means of questionnaires. As a possible future study, it seems interesting to compare players’ and coaches’ perceptions of their motivational styles, and to examine their relationship with BPN satisfaction and frustration, as well as players’ sport commitment. The use of observational methodology to examine coaches’ motivational behaviours may be also of great use in triangulating the results. Finally, the present study adopted a variable-centred approach and, therefore, did not consider whether coaches were able to combine autonomy-supportive and controlling behaviours. Future person-centred studies should examine whether coaches can simultaneously use both interpersonal styles [49], as well as their relationship with athletes need-based experiences and sport commitment. 

## 5. Conclusions

The present study suggests how the autonomy-supportive style provided by the coach may play a key role not only in satisfying the BPN and optimising the sport commitment of youth soccer players but also in avoiding BPN frustration. Likewise, the controlling style adopted by the coach, especially intimidation and the controlling use of rewards, in addition to facilitating the frustration of players’ BPN, may also reduce BPN satisfaction, greatly affecting the motivational process of young athletes. In this sense, it seems important to develop training strategies for coaches of youth soccer players that allow them to maximise autonomy-supportive behaviours and move away from controlling behaviours both in training and in competition. In this way, soccer players will experience positive motivational outcomes, which will result in greater sport commitment. All of this could help to reduce the high number of cases of sport dropouts in young soccer players. 

## Figures and Tables

**Table 1:** Descriptive statistics, reliability coefficients, and Pearson’s correlations between study variables.

Variables	*Range*	*M*	*DT*	ω	1	2	3	4	5	6	7	8
1. Autonomy support	1–7	5.59	1.00	0.92	−							
2. Controlling use of rewards	1–7	2.50	1.28	0.80	−0.135	−						
3. Negative conditional regard	1–7	2.60	1.32	0.82	−0.396 **	0.596 **	−					
4. Intimidation	1–7	2.33	1.22	0.79	−0.379 **	0.537 **	0.722 **	−				
5. Excessive personal control	1–7	2.10	1.12	0.68	−0.256 **	0.482 **	0.569 **	0.633 **	−			
6. BPN satisfaction	1–5	3.87	0.47	0.89	0.529 **	−0.169 *	−0.286 **	−0.306 **	−0.192 **	−		
7. BPN frustration	1–7	2.54	1.15	0.92	−0.453 **	0.412 **	0.612 **	0.646 **	0.503 **	−0.508 **	−	
8. Sport commitment	1–5	4.11	0.63	0.71	0.448 **	−0.242 **	−0.324	−0.305 **	−0.153 **	0.622 **	−0.350 **	−

Note: ω = McDonald’s omega; ** *p* < 0.01, * *p* < 0.05.

**Table 2:** Indirect effects of coach autonomy support, controlling use of rewards and intimidation on sport commitment.

	*Standardised estimates β*	Standard Error	*p*-Values
*Indirect effects of autonomy support on sport commitment*			
Total indirect	0.13	0.04	0.001
Specific indirect via BPN satisfaction autonomous motivation	0.04	0.02	0.038
Specific indirect via BPN frustration motivation	0.09	0.04	0.009
*Indirect effects of controlling use of rewards on sport commitment*			
Total indirect	−0.08	0.06	0.198
Specific indirect via BPN satisfaction autonomous motivation	0.02	0.02	0.296
Specific indirect via BPN frustration motivation	−0.10	0.05	0.043
*Indirect effects of intimidation on sport commitment*			
Total indirect	−0.27	0.10	0.006
Specific indirect via BPN satisfaction autonomous motivation	−0.18	0.09	0.037
Specific indirect via BPN frustration motivation	−0.09	0.05	0.057

## Data Availability

The data presented in this study are available on request from the corresponding author.
